# Perspective: Implications of the COVID-19 Pandemic for Family-Focused Practice With Parents With Mental Illness and Their Families

**DOI:** 10.3389/fpsyt.2022.806550

**Published:** 2022-04-11

**Authors:** Angela Obradovic, Joanne Nicholson

**Affiliations:** ^1^Northern Area Mental Health Services, Melbourne, VIC, Australia; ^2^The Heller School for Social Policy and Management, Institute for Behavioral Health, Brandeis University, Waltham, MA, United States

**Keywords:** family-focused practice, parents with mental illness, children of parents with mental illness, pandemic, adaptations, COVID-19

## Abstract

The goal of this perspective piece is to suggest challenges to family-focused practice with parents with mental illness and their children that have emerged during the COVID-19 pandemic. We discuss implications for practice, policy, and research that will benefit from rigorous study in the future, as we sift through lessons learned. The impact of the pandemic on the mental health and well-being of people around the world has been documented. Common adaptations in service delivery have included a shift to telehealth and digital tools. The pandemic has posed challenges to practice across the EASE Framework components for family-focused practice (i.e., Engage, Assess, Support, and Educate) for both parents/families and practitioners. Governmental policy and funding responses will be critical to addressing the impact of stresses, disruptions and losses endured during the past months. Pandemic experiences and consequences have implications for research measures, methods, and outcomes, given the dramatic changes in people's lives and the contexts in which they live. The shift to research implementation in virtual environments has resulted in challenges in maintaining confidentiality, and the privacy and security of data. As we move forward, it will be important to acknowledge the remaining uncertainty about the future and accommodate the profound changes in family life, professional practice, and research implementation related to the pandemic in our efforts to demonstrate the effectiveness of positive lessons learned while developing new approaches for dealing with the negative consequences of the pandemic.

## Introduction

Over the past few decades, the practice, lived experience, and research communities have engaged in ongoing discussion to specify core principles of family-focused practice aimed at improving outcomes for parents experiencing mental illness and/or substance use disorders and their children. Consensus has emerged across diverse cultural and systemic settings where stakeholders collaborated to facilitate local adaptation of evidence-based interventions (1–11). Practice principles underpinning these initiatives include the importance of asking about an adult's parenting and family status, or a parent's mental health and well-being when children are being seen. Treatment and recovery are viewed as relational and multidirectional processes, with families providing the context for change. Relationships with parents and family members are built on trust and a non-judgmental, trauma-informed approach. Family roles, responsibilities, needs, and resources are assessed and considered in care planning and goal setting, leveraging the strengths of parents and children to address vulnerabilities. The coordination and integration of supports across diverse service sectors and natural networks are advised, in the context of a collaborative, therapeutic partnership approach. Psychoeducation about mental health benefits both parents and children (4, 5, 11–15).

Any changes, temporary or otherwise, in the biopsychosocial challenges families face and the factors that mediate risk, enhance resilience, and support personal and relational recovery require re-examination in light of the COVID-19 global pandemic (16). A contemporaneous understanding of the pivotal elements of socio-cultural and service system contexts is paramount to ensuring that future practice, policy, and research in this field remain evidence-based, and that lessons learned during and due to the pandemic are incorporated into our efforts.

In this perspective piece, we lay out a range of possible impacts of the COVID-19 pandemic on the core elements of family-focused practice. We borrow the EASE Framework (4), a theory- and evidence-informed practice approach to relational recovery for parents with mental illness, to organize our comments for this perspective. Our discussions with mothers with mental and substance use disorders and practitioners over the past 2 years further inform our consideration of the ways in which the pandemic has challenged family-focused practice.

As experienced clinicians and researchers in two different countries, cultures, and service contexts, we recognize that our perspectives are shaped by our own lived experience of the pandemic and the lives, experiences, and stories of those around us. Our goal is not to provide recommendations based on research findings but, rather, to suggest challenges and discuss implications for family-focused practice, policy, and research that will benefit from rigorous study in the future, as we sift through lessons learned to endorse positive consequences of the pandemic and move past negative impacts.

## The Pandemic has Challenged Family-Focused Practice

The impact of the pandemic on the mental health and well-being of people around the world has been documented (17–20). Common adaptations in service delivery have included a shift to telehealth and digital tools (21). The pandemic has posed challenges to practice across the EASE Framework components: Engage, Assess, Support and Educate (4). Challenges have differed somewhat, depending on family circumstances and whether parents and families are newly referred or have existing relationships with practitioners (16).

### Engage

Engagement is the process of building a relationship with a parent and relevant family members, establishing rapport and trust to promote further collaboration (4). The pandemic has disrupted traditional, in-person modes of contact for practitioners with parents and families in many ways. Emerging, increasingly routine solutions (e.g., video conferencing) have necessitated attention to building and strengthening relationships within new arrangements. Cognizant of the limitations of virtual contact, practitioners have had to adapt or seek new ways to build trust, develop rapport, and maintain the safe, collaborative therapeutic partnerships essential to family-focused practice (22).

Safety imperatives have curtailed face-to-face contact by suspending home visiting or changed how this might occur due to the need for PPE protection, physical distance and, where possible, outdoor exchange. The focus of all parties (i.e., parents, families, practitioners, and programs) on minimizing the threat of infection have fundamentally altered the ways and locations in which parents, families and practitioners connect. Staying mindful of family needs and promoting connection likely have been challenging within some settings (e.g., hospital emergency departments or adult psychiatry inpatient units). Fundamental physical safety priorities have curtailed family visiting and possibly contributed to the breakdown of inter-team care coordination. During a crisis of this proportion, staff may have been redeployed away from their usual duties, potentially disrupting established therapeutic relationships.

While digital technology enables virtual connection as an alternative to face-to-face contact, these modes are not without disparity in their availability, cost, quality, capacity, and user proficiency. They also largely create a de facto incursion into the home, with potential privacy issues, complicating engagement in a way that office-based visits, if they were the norm, do not. The presence of children and other household members who may not be the focus of the session may affect levels of comfort, openness to sharing, concentration, and focus. Where sensitive matters such as abusive relationships, substance use or issues undisclosed to others in the household exist, virtual home-based sessions can be problematic. Practitioners may be exposing their own households to the parents they work with in virtual sessions, in ways that may inhibit or, alternatively, promote discussions of children and family life.

### Assess

Assessment involves asking key questions in the context of the parent-practitioner relationship (4). The pandemic may have mixed impact on assessment of parents and family members, particularly in new relationships. If assessments are done virtually, practitioners may be concerned about missing the cues or signals that inform clinical judgement. Peer recovery coaches, in our community engagement discussions, shared their concerns about possibly missing signs of substance use relapse that typically informed their “hunches” about how parents were doing.

Virtual sessions may provide glimpses into the parent's home and family life, previously unavailable. Parents may face the disclosure of information—as seen by the practitioner or overheard by the child or partner—they might not have provided in an initial assessment session. Parents may say more than they might have typically offered in the past, as being stressed and feeling frustrated, unhappy, or anxious have become commonplace in the COVID-19 context. Articles in the popular press highlight “parenting burnout” and underscore the stress accompanying remote learning for children due to lockdown. The pandemic may have, in some ways, given parents permission to openly discuss their challenges and ask for help, in a less judgmental context and with fewer negative consequences.

In many ways, family life has changed significantly. Routine assessment probes such as the request to “describe a typical day” have new meaning when days are no longer “typical” and a “new normal” is emerging over time. Life may have slowed down or become more intense, as obligations and commitments outside the home have been limited by travel restrictions and family members are spending more time together. When coping (e.g., with the threat of illness exposure or pandemic-induced isolation) is perceived as challenging for everyone, practitioners' assessment of risk or the identification of family members' strengths may be altered.

Parents' resilience and coping strategies may be enhanced by opportunities for practice or overwhelmed by stresses conveyed by the pandemic. The “mom-wine” culture touted in social media may well have influenced alcohol consumption over the past months, as parents cope with unprecedented pressures through increased substance use (23). Clearly, a non-judgmental, accepting stance on the part of the practitioner is warranted, with appropriate questions posed to clarify any assumptions made during an assessment.

### Support

Support includes helping parents take steps toward realistic goals, to achieve their vision for the family (4). The provision of instrumental or emotional support directly, or via the sharing of information and connections or referrals to resources have likely been significantly impacted by the pandemic. Exploring immediate unmet needs has been complicated both by the awareness that fewer solutions are available, and that methods of access to them have been hampered. On the other hand, a parent may be more likely to identify needs that have become more readily apparent due to the pandemic, as people's awareness and acceptance of challenges in day-to-day living have become the norm.

Developing and bolstering natural and professional support networks for parents, their children, partners, and other family members—a key activity in family-focused practice—may well be a major challenge. The ability to establish crisis and family care plans may be undermined by the lack of access to the established safety net of the extended family, respite resources, and school or childcare programs. In some cases, the pandemic may have led to a permanent loss of kinship care options, particularly affecting proactive parent-led prevention of relapse, or planning for hospitalization when alternative care options are vital. A fear that hospitalization may expose an individual and, therefore, family to infection may have led to avoidance of help seeking or planned admission, undermining a parent's recovery. In addition, demands on hospitals to focus on the acute needs of COVID-19 patients, has made in-patient care harder to access for those with non-COVID-19 related concerns.

The safety valve and empowerment provided by peer support and group programs for parents and children may have also been diminished through cancellation, postponement, or transfer to online formats. Online platforms have allowed some continuity of support, but the effectiveness of online approaches may vary depending on the resources and technical capabilities of users and services. The capacity to refer to additional professional help for individuals or whole families (e.g., counseling, infant and maternal mental health, family therapy) may have been limited by demands on the workforce to serve a much larger proportion of the population who have been affected by the pandemic.

### Educate

The provision of evidence-based psychoeducation to parents and children is a key tenant of family-focused practice (4). The pandemic has resulted in an “info-demic” as parents and families are bombarded by health information, constantly changing as new research findings emerge. The pandemic has placed mental health center stage, with more information available in the popular press than ever before (e.g., suggestions for recognizing depression, coping with anxiety, etc.). Health literacy skills have become increasingly important, as parents and families make decisions about whether and when to seek help for emotional and behavioral problems, along with pandemic-specific decisions about social distancing, mask wearing and vaccination. Parents may require or request assistance in sifting through available information.

Psychoeducation about coping with loss and grief has become increasingly important. Families may have lost family members to illness and death; lost regular contact with extended family and friends; and lost the normal routines that comprised pre-pandemic family life. Continuous proximity of family members without sufficient relief during lockdowns has for many, intensified relationships within the home and resulted in emotional dysregulation, relational strain, and interpersonal hostility (16). The practitioner may be faced with the challenge of teasing apart the impact of the pandemic and trauma conveyed from normal development issues, though the parent and family's needs and the professional's response may be similar in either case. The frame placed on the issues is critical in supporting parents and families in drawing upon their strengths, rather than succumbing to their vulnerabilities.

## Discussion: Future Implications

The challenges to family-focused practice during this period of pandemic crisis require us to consider the future implications for families, communities, and the workforce. This Discussion highlights opportunities to strengthen core principles, to identify potential undermining of advances made to date, and to refresh the practice and research agendas for the future.

### Families and Communities

As time passes, more detailed evidence of the impact of the COVID-19 pandemic is emerging, confirming expectations that the incidence of mental ill health has indeed increased, with females and younger age groups particularly affected by depressive and anxiety disorders (24). The social determinants of mental ill health are clearly reflected in the social and economic consequences of the pandemic and, in turn, to an exacerbation of pre-COVID mental illnesses. This is of particular concern in families where parents had prior mental or substance use disorders and find their ability to manage their illnesses and cope with the demands of childrearing overwhelmed by pandemic-related stresses.

However, while generating significant demand for mental health services, this increase in higher prevalence psychiatric disorders (e.g., depression, anxiety) and greater awareness of the multidimensional, interdependent nature of mental distress and adversity across the lifespan, also create opportunities to:

- lessen the stigma, shame and secrecy that often accompany mental ill health or substance use;
- normalize open discussion about mental health or substance use concerns and, therefore, encourage help-seeking behavior;
- mobilize previously untapped social support networks (e.g., community and neighborhood opportunities), particularly for children and adolescents (25);
- increase recognition of the need for co-design in planning sustainable local responses (26, 27);
- gain public and governmental support for addressing mental health and substance use challenges, overcoming policy-related barriers to services and increasing funding for prevention, early intervention, and treatment.

What the post-pandemic context holds for those affected by lower prevalence mental illnesses typically considered more serious (e.g., schizophrenia, bipolar disorder), either pre-existing or newly onset, is less clear. Given the historical failure to implement and scale up adequate responses to those living with mental ill health despite compelling evidence of effective interventions, it may be the case that this heightened public awareness will steer discourse, service orientation, and publicly funded resources and research away from serious and enduring mental illnesses to the more commonly experienced situational, adjustment, and higher prevalence psychiatric disorders.

### Workforce

One of the implications of this much broader experience of adversity and crisis is the effect on practitioners (28, 29). Many family-focused practitioners have undoubtedly encountered significant stress in their personal and professional lives, which may have generated reflection and reappraisal. Career reassessment is occurring in health care fields and in the workforce in general (30). Future inquiries should examine the extent of pandemic-related secondary impacts on family-focused practitioners of trauma, helplessness, compassion fatigue, and work-related moral injury (i.e., the profound psychological distress that comes from actions or the lack of, which violate one's moral or ethical code) (31).

If the emotional reserves of professionals have been tested, how has that impacted therapeutic relationships? Are practitioners more attuned to their parent-clients and the needs of the children in these families (32)? In training and supervision, practitioners may be able to draw from their personal exposure to adversity to consider their perception of risk and their capacity to maintain a fundamentally non-judgmental, strengths-based approach in their work. With the significant community effort and experience in collective caring and interpersonal sharing during this crisis, we also have an opportunity to elevate the utility of both peer support and recovery approaches across health and social care sectors, given their natural alignment to pandemic responses and healing.

If we accept that reliance on digital platforms has been embraced as an acceptable practice adaptation, this could lead to framing Internet access as a basic right, requiring future mental health service delivery to maintain a flexible menu of virtual and face-to-face options tailored to individual need, preference, and suitability. This could include:

- peer group programs that consist of a hybrid combination of face-to-face and online sessions;
- home visiting prioritized for individuals and families without reliable digital access;
- face-to-face interaction prioritized where virtual contact interferes with micro-communication; and
- increase in the available workforce by including those who cannot undertake a full time, site based or home visiting role due to other commitments or restrictions (e.g., digital service delivery by those home-bound).

It would be wise for the practice community to prepare actively to advocate against the repetition of past experience. Competing priorities in any service reconfiguration because of the pandemic may mean the needs of parents with mental illness and their families go unrecognized or untreated, as has occurred historically when resources are scarce.

### Research

Our pandemic experiences have implications for research measures, methods, and outcomes. Treatment targets and timeframes may have shifted, given changes in modes of engagement, contextual stresses, and coping strategies. The validity and reliability of research measures routinely employed to assess variables and outcomes may need to be re-evaluated, given the dramatic changes in people's lives and the contexts in which they live. For example, traditional measures of social networks, social support, social functioning, self-efficacy, family functioning and family relationships may require careful examination, given the likely impact of the pandemic at a population level on each of these. Intervention fidelity measures, possibly based on frequency or type of visits, may need to be adjusted to take virtual encounters into consideration.

The pandemic has likely had positive and negative impacts on research practices. The shift to virtual encounters has, in some ways, made research interviewing more streamlined and expeditious by eliminating the need for travel. On the other hand, issues of privacy and confidentiality have been raised, as both the participant and the researcher are more exposed on the video screen. The role and responsibilities of institutional review boards have become more complex, as issues of data collection, management, and security have been challenged by requirements for limited in-person contact and social distancing. Research with parents and families may have been limited during this time to those with access to the Internet and necessary equipment. Alternatively, the shift to virtual data collection may have resulted in the ability to access target populations previously untapped or more diverse than in-person data collection permitted.

## Conclusion

In conclusion, we acknowledge that our perspective is based on our experiences as clinicians and researchers in two well-resourced, first world countries. Our goal is to bring attention to both the challenges and the opportunities the pandemic brings to family-focused practice, and the parents, children, and families with whom we partner. As we move forward, it will be important to acknowledge the remaining uncertainty about the future and accommodate to the profound changes in family life, professional practice, and research related to the pandemic, as we demonstrate the effectiveness of positive lessons learned while developing new approaches for dealing with negative consequences.

## Data Availability Statement

The original contributions presented in the study are included in the article/supplementary materials; further inquiries can be directed to the corresponding author/s.

## Author Contributions

AO and JN were jointly responsible for the development and preparation of the manuscript. All authors contributed to the manuscript, read, and approved the submitted version.

## Funding

Community engagement activities serving as a source for this article were funded through two Patient-Centered Outcomes Research Institute^®^ (PCORI^®^) Awards: #EAIN-00147 and #8285-BU.

## Author Disclaimer

The views and statements presented in this article are solely the responsibility of the authors and do not necessarily represent the views of the Patient-Centered Outcomes Research Institute^®^ (PCORI^®^), its Board of Governors or Methodology Committee.

## Conflict of Interest

The authors declare that the research was conducted in the absence of any commercial or financial relationships that could be construed as a potential conflict of interest.

## Publisher's Note

All claims expressed in this article are solely those of the authors and do not necessarily represent those of their affiliated organizations, or those of the publisher, the editors and the reviewers. Any product that may be evaluated in this article, or claim that may be made by its manufacturer, is not guaranteed or endorsed by the publisher.
